# A Pilot Study on the Efficacy of a Diabetic Diet Containing the Rare Sugar D-Allulose in Patients with Type 2 Diabetes Mellitus: A Prospective, Randomized, Single-Blind, Crossover Study

**DOI:** 10.3390/nu15122802

**Published:** 2023-06-19

**Authors:** Kensaku Fukunaga, Takafumi Yoshimura, Hitomi Imachi, Toshihiro Kobayashi, Takanobu Saheki, Seisuke Sato, Nao Saheki, Wenyi Jiang, Koji Murao

**Affiliations:** Department of Endocrinology and Metabolism, Faculty of Medicine, Kagawa University, 1750-1, Miki-cho, Kita-gun 761-0793, Kagawa, Japan; fukunaga.kensaku@kagawa-u.ac.jp (K.F.); yoshimura.takafumi.j4@kagawa-u.ac.jp (T.Y.); imachi.hitomi@kagawa-u.ac.jp (H.I.); kobayashi.toshihiro@kagawa-u.ac.jp (T.K.); saheki.takanobu@kagawa-u.ac.jp (T.S.); seisuke.310.med@gmail.com (S.S.); saheki.nao@kagawa-u.ac.jp (N.S.); jiang.wenyi@kagawa-u.ac.jp (W.J.)

**Keywords:** D-allulose, rare sugar, diabetic diets, isCGM, postprandial blood glucose levels

## Abstract

High sugar consumption increases the risk of diabetes, obesity, and cardiovascular diseases. Regarding the diet of patients with diabetes, artificial sweeteners are considered a safe alternative to sugar; however, there is also a risk that artificial sweeteners exacerbate glucose metabolism. D-allulose (C-3 isomer of d-fructose), which is a rare sugar, has been reported to have antidiabetic and antiobesity effects. In this study, the efficacy of a diabetic diet containing D-allulose was investigated in patients with type 2 diabetes using an intermittently scanned continuous glucose monitoring system (isCGM). This study was a validated, prospective, single-blind, randomized, crossover comparative study. Comparison of peak postprandial blood glucose (PPG) levels after consumption of a standard diabetic diet and a diabetic diet containing 8.5 g of D-allulose was the primary endpoint. A D-allulose-containing diabetic diet improved PPG levels in type two diabetes patients compared with a strictly energy-controlled diabetic diet. The results also showed a protective effect on endogenous pancreatic insulin secretory capacity owing to reduced insulin requirement. In patients with type two diabetes mellitus, diabetic diets containing 8.5 g D-allulose were effective in improving PPG levels.

## 1. Introduction

Statistics released by the International Diabetes Federation in 2021 [1] reported that the global population with diabetes is estimated to be 537 million, with approximately 1 in 10 adults (10.5%) suffering from this disease. Without effective countermeasures, the diabetic population is expected to reach 643 million (11.3%) by 2030. Furthermore, it is projected to increase to 783 million (12.2%) by 2045, indicating an alarmingly rapid increase in diabetes worldwide. More than 90% of people with diabetes worldwide have type two diabetes as a cause of diabetes mellitus. Type two diabetes begins with a lack of insulin action caused by decreased insulin secretion and insulin resistance. The basis of treatment for type 2 diabetes includes diet, exercise, and drug therapy; however, the most important of these treatments is the improvement of lifestyle habits such as diet and exercise.

The risks associated with high dietary sugar intake have been widely reported in recent years. Obesity, diabetes, and cardiovascular diseases etc., are closely related to excessive sugar intake [2,3], and there is growing interest in safe alternative sweeteners that can replace sugar. It has been suggested that the use of artificial sweeteners in place of sugar may lead to the prevention and treatment of diseases such as diabetes and obesity [4]. On the other hand, the potential risks of artificial sweeteners on glucose metabolism have also been reported in recent years [5,6,7,8].

Monosaccharides, which are the smallest functional units of carbohydrates, are widely present in nature as natural monosaccharides. These include substances such as D-glucose and D-fructose. In contrast, “rare sugars” are generic terms for sugars and their derivatives that exist only in very small quantities in nature [9]. D-allulose has a sweetness of about 70% of sugar. In the small intestine, approximately 70% of the D-allulose ingested in the body is absorbed and circulates throughout the body, but more than 99% is excreted in the urine [10]. In this process, it has been suggested that its intestinal absorption and renal excretion are mediated by transporters. Hishiike et al. [11] reported that during this process, D-allulose may be taken up by intestinal cells from the intestinal lumen via GLUT5 and transported to the capillary via GLUT2. Kishida et al. [12] also reported that GLUT5 mediates D-allulose transport in the gut. In addition, they reported the possibility that SGLT1 may be involved in mediating the uptake of D-allulose. As for the fermentability of d-allulose, D-allulose that is not absorbed in the small intestine is transferred to the large intestine, but in humans it has been shown to be less fermentable by intestinal bacteria in the large intestine [13]. It has also been reported that unfermented D-allulose is rapidly excreted in feces, with 8% to 13% of the dose recovered within the first 24 h [14]. Several anti-diabetic and anti-obesity effects of rare sugars have been reported in basic research and clinical studies. As an inhibitory effect on monosaccharide absorption, it has been reported that D-allulose inhibits the transport and absorption of D-fructose and D-glucose in competition during the absorption phase from the gastrointestinal tract [11]. This is thought to compete with the process by which they are taken up by intestinal cells from the intestinal lumen of the small intestinal mucosa and transported to the capillaries. In addition, improvements in insulin secretion capacity and pancreatic protection in pancreatic beta cells have also been reported [15]. When OLETF rats (type two diabetes model rats) were treated with D-allulose and observed for 60 weeks, glucose tolerance was improved, body weight gain was reduced, and insulin secretion capacity was maintained in the allulose-treated group. Pathological findings also showed that the pancreatic islet of Langerhans structure was maintained in the D-allulose group. Cytokines were also examined, and various inflammatory markers, such as TNF-α and IL-6 were significantly reduced. These results suggest that D-allulose may be beneficial for improving glucose tolerance with β-cell protection and for anti-obesity effects. It has also been reported that when mice were fed D-allulose orally, the secretion of glucagon-like peptide-1 (GLP-1), which is one of the major gastrointestinal hormones, such as cholecystokinin and peptide YY, was accelerated. GLP-1 had a suppressive effect on food intake via the afferent vagus nerve and improved glucose tolerance by enhancing insulin sensitivity in a blood glucose-dependent manner [16].

Clinical trials have reported its efficacy in suppressing PPG levels after glucose loading and test meal consumption in healthy adults [13] and adults with impaired glucose tolerance [17]. We have previously studied the effect of D-allulose consumption during the fasting period of Ramadan on PPG levels in Muslims with type two diabetes [18]. PPG levels were studied using an intermittent continuous glucose monitoring system (isCGM), and D-allulose significantly reduced PPG levels in patients with type two diabetes mellitus. Although various studies have been conducted in this manner, there are no reports on the effects of using D-allulose as a therapeutic diet for diabetes treatment, which is the most important aspect of diabetes treatment. In this present study, we report a detailed investigation of the efficacy of a therapeutic diet containing the rare sugar D-allulose in patients with type two diabetes using isCGM data.

## 2. Methods

### 2.1. Study Design

**Figure 1:**
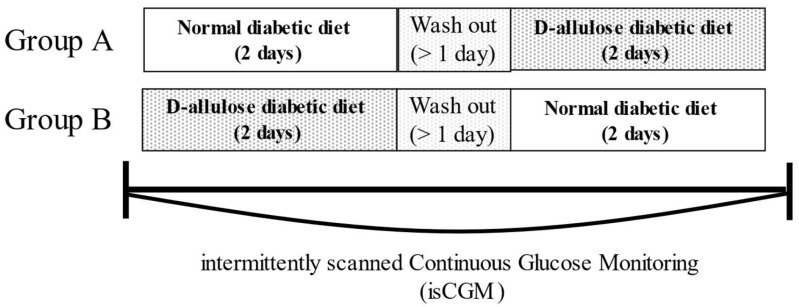
Schematic representation of the study design. Group A: Diabetic diet prior group. Group B: D-allulose-containing diabetic diet prior group.

This study was a validated, prospective, randomized, single-blind, crossover, comparative study (single-blinding was carried out in participants). In the present exploratory study, we designed a crossover comparative study comparing the intervention and control groups in the same subjects in order to conduct a detailed study with a limited number of cases. We took into account that this method has the advantage that the effects of individual differences can be properly assessed. On the other hand, as a precaution for crossover comparative trials, we planned a statistical analysis of the washout period using the method described below, although it was necessary to check for carryover effects. This study enrolled 24 patients who were admitted to Kagawa University Hospital between December 2019 and August 2022. Patients were randomly divided into groups A and B. The allocation of study subjects to each group was carried out using the envelope method. Between these two groups, blood glucose levels during the study period were measured using the isCGM device FreeStyle Libre (software version 2.2.13,0.94, Abbott Japan Diabetes Care Inc., Tokyo, Japan) (Figure 1).

Group A: Diabetic diet preemptive group. Diabetic patients in the hospital were fed a diabetic diet three times daily for two days. After a washout period of at least one day, a diabetic diet containing D-allulose was provided three times a day for two days.

Group B: D-allulose-containing diabetic diet prior group. Diabetic patients in the hospital were fed a diabetic diet containing D-allulose three times daily for two days, followed by a washout period of at least one day and a diabetic diet three times daily for two days.

The D-allulose content in the rare sugar-containing diabetic diets used in this study was 8.5 g D-allulose per meal. The medications used prior to the study were continued during the study. The number of insulin units was not adjusted, nor were oral hypoglycemic drugs added, changed, or dose adjusted during the study period. We obtained prior written informed consent from all participants prior to enrolment in the study. The study was conducted in compliance with the Declaration of Helsinki (latest version) of the World Medical Association and the Japanese Clinical Research Act. This study was approved by the Clinical Research Review Committee of Kagawa University Hospital (approval number: CRB6200005, Recognition date: 29 September 2020). This study was registered with the Japan Registry of Clinical Trials (trial registration number: jRCTs061200022).

In the study, the patients participated over a period of five days. During this period, blood glucose levels were measured using isCGM, which consisted of a two-day period on a regular diabetic diet, a washout period of at least one day, and a two-day period on a diabetic diet containing D-allulose.

### 2.2. Participants

The main selection criteria for patients were as follows: age between 20 and 80 years, HbA1c ≥ 6.5%, hospitalized type two diabetes patients, and documented consent to participate in the study. The main exclusion criteria were as follows: patients taking alpha-glucosidase inhibitors, patients with a history of serious adverse reactions to D-allulose in the past, and patients with serious complications or serum creatinine ≥1.5 mg/dL (severe renal dysfunction). Pregnant, parturient, lactating, or possibly pregnant women; patients participating in other studies; or those deemed by their physician to be inappropriate for study participation were also excluded.

### 2.3. Study Food

Diabetic diets containing rare sugars contain 8.5 g D-allulose per serving. In previous reports, it has been reported that D-allulose can be expected to suppress postprandial blood glucose levels when 5 g or more is ingested with a meal or during a glucose tolerance test [13,17,19,20]. Iida et al. [21] reported that the maximum no-observed-effect level in humans was 0.55 g/kg body weight when the laxative effect of D-allulose was used as an indicator. In addition, in a safety report by Han et al. [22], the maximum single intake of D-allulose was 0.4 g/kg BW and the maximum total daily intake was 0.9 g/kg BW. Based on these reports, and with more emphasis on safety, the maximum no-observed-effect level for an assumed 50 kg body weight adult was 27.5 g (50 kg body weight × 0.55 g/kg body weight = 27.5 g), and a daily dose of 25.5 g (8.5 g × 3 meals = 25.5 g) that would not exceed this was set as the daily dose in this study. The energy intake per day for each diabetic meal was determined in accordance with the method for calculating the total energy intake (kcal/day) (target body weight [kg] × energy coefficient [kcal/kg]) recommended by the Japan Diabetes Society in its Guidelines for Diabetes Care 2019 [23]. The standards for the percentage of nutrients in the diet are also met: 50–60% carbohydrate, approximately 20% protein, and the remainder fat, respectively. The diabetic diet containing rare sugars simply added 8.5 g of D-allulose to the diabetic diet and had the same total calories as the regular diabetic diet. D-allulose was provided by Matsutani Chemical Industry Co., Ltd. (Hyogo, Japan) and RareSweet Co., Ltd. (Kagawa, Japan). Diabetic diets and diets containing rare sugars were prepared and supplied by Bosco Food Service Co., Ltd. (Kagawa, Japan).

### 2.4. Glycemic Measures

Blood glucose measurements were performed using the isCGM FreeStyle Libre (Abbott Japan Diabetes Care Inc., Tokyo, Japan). The FreeStyle Libre is a medical device that can measure glucose levels in the interstitial fluid. The measurement interval is every 15 min and has a validity period of 14 days. A high correlation between interstitial fluid glucose levels and blood glucose levels has been reported [24]. The E-test TOSOH II (IRI) and the E-test TOSOH II (CPR) EIA kit (TOSOH Corporation, Tokyo, Japan) were used to measure insulin immunoreactivity (IRI) and C-Peptide immunoreactivity (CPR).

### 2.5. Outcome Measures

The primary endpoint was the comparison of the peak PPG levels using isCGM. The secondary endpoints were the postprandial blood glucose trend when using isCGM, the proportion of time spent within the blood glucose target of 70–180 mg/dL (% Time In Range [TIR]), the proportion of time spent in the hyperglycemic range (>180 mg/dL) above the TIR (% Time Above Range [TAR]), and the proportion of time spent in the hypoglycemic range (<70 mg/dL) below the TIR (% Time Below Range [TBR]). C-Peptide immunoreactivity (CPR) before and after meals was analyzed, and the results of a questionnaire on patient satisfaction with seasoning and other factors were tabulated. Changes in the number of diarrheal episodes were identified as safety endpoints.

### 2.6. Statistical Analysis

With reference to previous studies [13], assuming an expected difference of −20 mg/dL, a standard deviation of the difference of 30 mg/dL, and α = 0.05 and power = 0.8 for the D-allulose-containing diabetic diet, the required sample size would be 20 cases if the correlation coefficient between pre- and post-values were hypothetically 0.5. To account for the small number of dropouts, the target number of participants was set to 24. In the main analysis, linear mixed-effects models were used with group, timing, and diet (normal diabetic diet vs. diabetic diet containing D-allulose) as fixed effects and individually as a variable effect on the peak postprandial blood glucose levels. A linear mixed-effects model was chosen because it analyses correlations between repeated measurements in the same subject and allows the evaluation of timing and carry-over effects. The null hypothesis was that the mean difference in peak postprandial blood glucose levels after each diet was zero, the *p* values were determined, and interval estimation was performed. The two-tailed significance level was set at 5%, and 95% confidence intervals were calculated. To assess the carryover effect, *p* values for the hypothesis test were determined (null hypothesis: the difference in peak postprandial blood glucose levels between the groups was zero), and interval estimates were performed. A linear mixed-effects model was also used for the analysis of the secondary endpoints. The significance level was set at 5% two-sided, and two-sided 95% confidence intervals were calculated. For the analysis of subject background, summary statistics of subject background data were calculated overall, by group (normal diabetic diet prior vs. D-allulose-containing diabetic diet prior), and by time point. The frequencies and proportions of the categories are presented for nominal and ordinal variables. For continuous variables, appropriate summary statistics (mean, standard deviation, median, and range, whichever was appropriate) were calculated. Between-group comparisons of background factors were not performed. Data for each treatment group are presented as the mean ± SEM. * *p* < 0.05 and ** *p* < 0.01 vs. normal diabetes diet group. All statistical analyses were performed using SPSS version 25 software (IBM Corp., Armonk, NY, USA).

## 3. Results

### 3.1. Participant Characteristics

**Figure 2:**
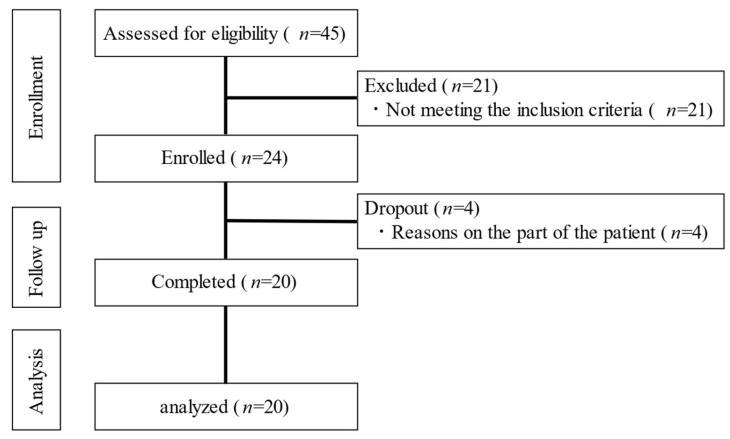
The flow chart of participant enrollment.

Figure 2 shows the chart of the enrollment. Among the 24 consenting cases, 4 cases were excluded; thus, the final analysis was performed in 20 cases (Figure 2). The participants’ age was 61.4 ± 12.1 years, HbA1c was 9.2 ± 1.8%, and duration of diabetes was 10.6 ± 11.5 years (Table 1). Table 1 shows the treatment details of each patient.

### 3.2. Effect of D-Allulose on Postprandial Peak Glucose

**Figure 3:**
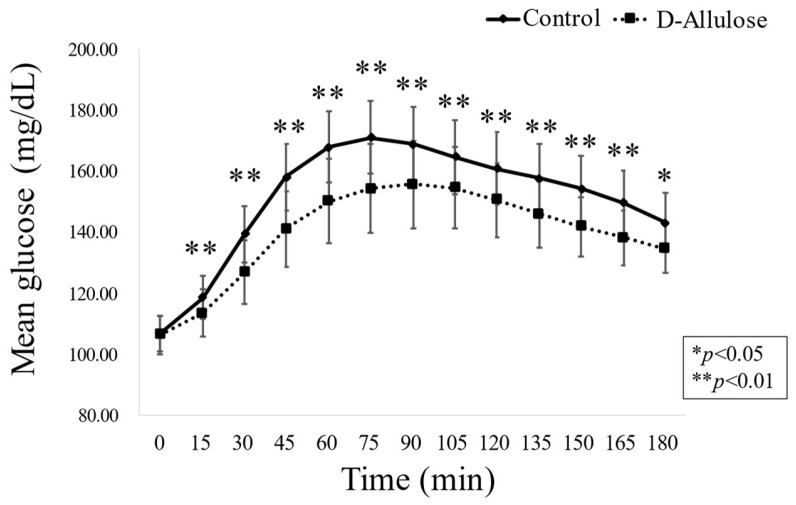

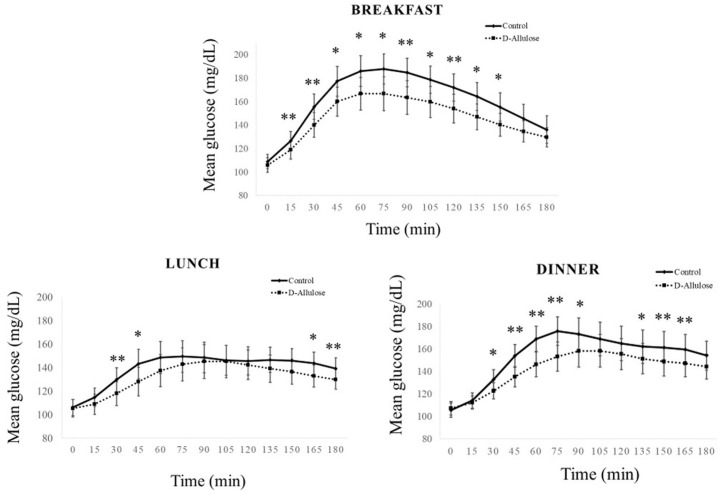
Mean glucose level curves for all meals and each meal during the intake period of each diabetic diet group. Blood glycemic variability at 0 (premeal) to 180 min after the meal in all 20 subjects is shown on the basis of isCGM data. The solid line shows glycemic variability with normal diabetic diet intake, and the dotted line shows glycemic variability with D-allulose-containing diabetic diet intake represents a statistically significant difference (* *p* < 0.05, ** *p* < 0.01, linear mixed-effects models) compared with the control group. Data reported as mean ± SEM.

As shown in Figure 3, the mean peak postprandial blood glucose level for each diet consumed was 173 mg/dL (95% confidence interval: 146, 200) for the diabetic diet containing D-allulose and 191 mg/dL (95% confidence interval: 163, 218) for the normal diabetic diet. The mean difference in the peak postprandial blood glucose levels was −18 (95% confidence interval: −24, −11; *p* value < 0.001). Therefore, the null hypothesis (the mean of the differences is zero) was rejected. This indicated that there was a statistically significant reduction in peak PPG levels with the D-allulose-containing diabetic diet compared with the normal diabetic diet. The carryover effect was also very small (estimate: −0.833; 95% CI: −38.72, 37.05; *p* = 0.965), and there were no problems with washout.

### 3.3. Postprandial Blood Glucose Levels When Using IsCGM

**Figure 4:**
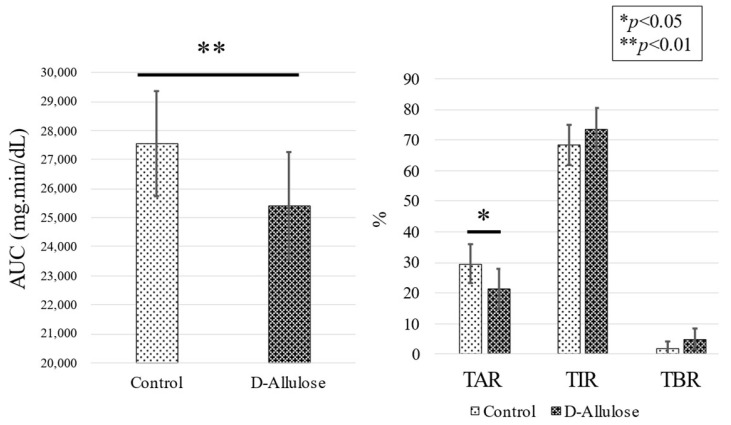
Comparison of AUC, %TIR, %TAR, and %TBR in the blood glucose variability curve. The AUC, TIR, TAR, and TBR from premeal to 180 min postmeal were analyzed. Represents a statistically significant difference (* *p* < 0.05, ** *p* < 0.01, linear mixed-effects models) compared with control group. Data are reported as mean ± SEM.

**Figure 5:**
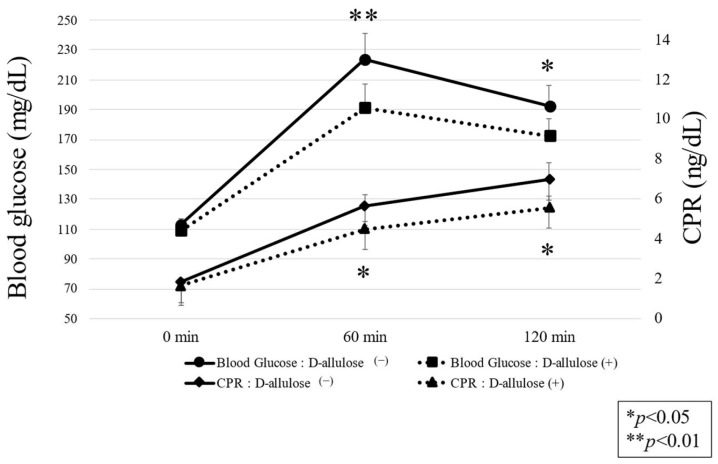
Comparison of postprandial endogenous insulin secretory capacity. Comparison of postprandial blood glucose and CPR levels after consumption of normal and D-allulose-containing diabetic diets. Represents a statistically significant difference (* *p* < 0.05, ** *p* < 0.01, linear mixed-effects models) compared with the control group. Data are reported as mean ± SEM.

The area under the curve (AUC) of 0 to 180 min postprandial blood glucose levels decreased (25,408 ± 1814 vs. 27,550 ± 1866 mg-min/dL. *p* < 0.001) and the ratio of %TAR also decreased (29.6 ± 6.4% vs. 21.4 ± 6.7%; *p* = 0.018) (Figure 4). There were no significant changes in the TIR ratio (68.3 ± 6.5% vs. 73.6 ± 6.8%; *p* = 0.211) or %TBR and no increase in hypoglycemia frequency (2.1 ± 2.1% vs. 5.0 ± 3.5%; *p* = 0.219). A detailed examination of blood glucose levels and CPR before and after meals showed that CPR decreased significantly as blood glucose levels decreased (Figure 5). These results suggest that a D-allulose-containing diabetic diet lowers postprandial blood glucose levels and, accordingly, insulin requirements.

### 3.4. Patient Questionnaire on Seasoning

Satisfaction in terms of quantity, seasoning, coloring, and smell was also ascertained (Table 2). The results for quantity (*p* = 0.6), seasoning (*p* = 0.322), coloring (*p* = 0.906), and smell (*p* = 1.00) showed no significant differences. There were no complaints of taste or odor being too sweet, and participants could not discriminate which was the rare sugar diabetic diet.

### 3.5. Side Effects of D-Allulose

No diarrhea or increased frequency of defecation was observed, and there were no safety concerns.

## 4. Discussion

This study is the first report on the use of rare sugars in a diabetic diet and the first detailed evaluation of blood glucose trends and endogenous insulin secretory capacity in patients with type two diabetes. This study was conducted in type two diabetes patients aged between 20 and 80 years old and compared the peak PPG levels when diabetic diets containing D-allulose were consumed using isCGM. The consumption of a diabetic diet containing 8.5 g D-allulose per meal improved PPG levels in type two diabetes patients even when compared with a strictly energy-controlled diabetic diet. Serum CPR levels also tended to decrease. In a previous report, 20 healthy adults ingested D-allulose during a 75 g oral glucose tolerance test (OGTT) to check blood glucose trends and insulin levels. This study found that 5 g or more of D-allulose was required to significantly suppress blood glucose levels after glucose loading [13]. Interestingly, blood insulin levels also decreased, which is consistent with our study results. Basic research has also reported a protective effect of D-allulose on pancreatic beta cells [15]. Hossain et al. reported protective effects on pancreatic tissue and the associated maintenance and improvement of insulin secretory capacity. These results suggest that D-allulose may be beneficial for improving glucose tolerance with β-cell protection. Given the results of reducing postprandial blood glucose levels without stimulating insulin secretion in the present study, it is suggested that D-allulose may contribute to correcting postprandial blood glucose levels and protecting pancreatic beta cells in type two diabetes patients with reduced insulin secretion capacity in clinical practice. However, additional studies with increased sample sizes and longer schedules are desirable in the future to verify this in more detail.

A systematic review and meta-analysis have also been reported [19]. Previous reports on control and D-allulose intake groups in healthy adults have been collected and compared, showing that 5 or 10 g of D-allulose resulted in significantly less area under the postprandial blood glucose curve. A study on subjects with impaired glucose tolerance (so-called borderline diabetics) has also been reported. When 15 glucose intolerant subjects consumed a test meal (84.5 g carbohydrate, 3.7 g fat, and 13.3 g protein: total 425 kcal) with a 5 g D-allulose load, PPG levels were significantly reduced [17]. Therefore, D-allulose improves PPG levels in healthy adults and borderline diabetic patients. Recently, we administered 8.5 g of D-allulose before Iftar to type two diabetes patients during Ramadan and examined its effect on postprandial glucose levels. D-allulose significantly suppressed postprandial hyperglycemia. In this study, D-allulose was added to three meals for patients with diabetes on admission, and a strict diabetic diet was used for comparison. These two hospital diets had the same calorie content and menu, but significantly suppressed postprandial hyperglycemia in type two diabetes patients.

Various mechanisms underlying the blood glucose-lowering effect of D-allulose have been reported. Basic research has reported the inhibitory effect of D-allulose on monosaccharide absorption in the small intestine [11], its effect on improving blood glucose levels by promoting glycogen synthesis via the activation of glucokinase in liver cells [25], its protective effect on pancreatic tissue, and the associated maintenance and improvement of insulin secretion capacity [15]. Its relationship with GLP-1 has also been reported [16]. Iwasaki et al. have examined the relationship between D-allulose and GLP-1 in detail. In an in vivo study, surgical transection of the afferent vagus nerve in mice abolished the effect of D-allulose administration on blood glucose levels. In addition, changes in food intake have also been investigated; severing the afferent vagus nerve abolished the appetite suppression effect of D-allulose administration. Furthermore, it was demonstrated that D-allulose activated the cell bodies of the afferent vagus nerve and the nucleus tractus solitarius of the medulla oblongata (projection site of the afferent vagus nerve), and that knockdown of GLP-1 receptor expression specifically in the afferent vagus nerve abolished the feeding suppressive effect of D-allulose. These results suggest that the mechanism of action of D-allulose may be to improve glucose tolerance and suppress appetite by promoting GLP-1 secretion and acting on the central nervous system via the afferent vagus nerve. While this may be one of the possible mechanisms of postprandial blood glucose reduction with D-allulose-containing diabetic diets in our study, some of the diabetic patients in this study were using GLP-1 receptor agonists. The use of GLP-1 receptor agonists provides pharmacological GLP-1 blood levels that far exceed the endogenous GLP-1 levels [26]. In the present study, the effect of blood glucose suppression after the consumption of a diabetic diet containing D-allulose was also observed in patients using GLP-1 receptor agonists. In view of this, it is very interesting to consider that other complex factors, in addition to GLP-1, are involved in the mechanism of action of D-allulose.

Owing to massive sugar consumption, alternative food has attracted considerable attention. Artificial sweeteners, which are frequently used as sugar substitutes, have been identified as potentially useful alternatives to sugar to prevent diabetes and obesity [4]. However, there are reports that artificial sweeteners may have adverse effects on glucose metabolism, and thus require caution. Previous large-scale epidemiological studies have shown an increased risk of obesity with increased consumption of artificially sweetened beverages [7]. This report did not examine eating habits, and while changes in eating behavior need to be fully considered, the possibility that the intake of artificially sweetened beverages may increase appetite, including impulsive eating, has also been reported [8]. These precautions should be taken into account when taking artificial sweeteners.

In this study, a diabetic diet was developed in the form of a normal therapeutic diet with the addition of D-allulose to unify the total energy content. There have been several reports on the properties of D-allulose when used in cooking. For example, with regard to changes in the viscosity of food products, previous studies on egg whites with various monosaccharides showed that the flow viscosity tended to decrease with increasing content when D-allulose was added, which was comparable to Fructose and Sucrose [27]. In addition, cookies cooked with D-allulose had increased antioxidants compared to the others. Based on these results, various studies on characterization are underway. When surveyed in patients, no negative opinions were found regarding seasoning or odors, such as sweetness. These findings have significant clinical implications. Various studies on what consumers want from sweeteners have also been conducted. Jürkenbeck et al. analyzed which characteristics of sweeteners are important to consumers for four sweeteners (D-allulose, stevia, xylitol, and erythritol), including D-allulose [28]. According to them, the most important factor for consumers when purchasing sweeteners was ‘taste’. Consistent with this report, it has been reported that taste was still an important consideration when purchasing sugar and sugar substitutes, in addition to the importance of whether the product is of natural origin [29]. On the other hand, there are also reports that consumers place more importance on the calorie content of food products, as labelling the calorie content of food products can lead to healthier food choices [30]. In any case, the results of this study suggest that D-allulose does not seem to have any tasting problems, is naturally derived, and low in calories, which is important evidence that a diet containing rare sugars could be widely accepted by many people in the future.

With regard to the safety of D-allulose, studies have shown that D-allulose can be safely consumed in healthy adults [21,22] and in type two diabetes patients [31]. Iida et al. [21] reported that the maximum no-observed-effect level in humans was 0.55 g/kg body weight when the laxative effect of D-allulose was used as an indicator. An investigation into the dosage of D-allulose was also carried out in a gastrointestinal tolerance study in healthy adults [22]. The results showed that 0.4 g/kg BW was the maximum single intake of D-allulose. The maximum total daily intake was also identified and reported to be 0.9 g/kg BW. In the present study, we used 8.5 g D-allulose per meal (total of 25.5 g per day). There were no safety issues, and this finding is consistent with previously reported results. No safety issues have been reported with regard to the long-term intake of D-allulose. Borderline and type two diabetic patients consuming 5 g of D-allulose three times a day with meals for 12 weeks had no safety problems.

The limitations of our study were as follows. First, this was an exploratory study, and the number of patients involved was small; therefore, the effect of D-allulose should be studied in a larger number of cases in the future. Second, we conducted this study in a controlled setting with patients in the hospital, and the duration of the study was short. Tak et al. [32] reported that the effects of oral dietary supplements containing D-allulose in type two diabetes patients over an eight-week period. According to a previous study, the intake of oral dietary supplements containing D-allulose improved glycemic profiles, such as fasting blood glucose, HOMA-IR, and HbA1c, and reduced BMI and body weight. For example, the effectiveness of the D-allulose diabetic diet in type two diabetes patients in daily life and further studies on its long-term consumption are needed. Resolving these limitations in the future will help to better understand the impact of D-allulose on diabetic patients.

In this study, a diabetic diet containing 8.5 g D-allulose was found to improve PPG levels in type two diabetics compared with a normal diabetic diet. This is the first report to show in detail that a rare sugar diabetic diet improves blood glucose levels in type two diabetes patients. On the basis of these results, further analyses will be possible in the future.

## Figures and Tables

**Table 1:** Clinical characteristics of the enrolled subjects with type 2 diabetes mellitus and diet composition of study foods. BMI, body mass index; BP, Blood Pressure; HbA1c, hemoglobin A1c; IRI, immunoreactive insulin; CPR, C-Peptide immunoreactivity; UACR, Urine Albumin-to-Creatinine Ratio; LDL, Low Density Lipoprotein; HDL, High Density Lipoprotein; BUN, blood urea nitrogen; Cre, creatinine; eGFR, estimated Glomerular Filtration Rate; AST, aspartate aminotransferase; ALT, alanine aminotransferase; CVR-R, Coefficient of variation of R-R interval; CAVI, Cardio Ankle Vascular Index; ABI, Ankle Brachial Index; OHAs, oral hypoglycemic agent; GLP-1RA, glucagon-like peptide-1 receptor agonists.

	Overall	Control First Group	D-Allulose First Group	*p*-Value
Gender				
Male	14 (70.0%)	6 (60.0%)	8 (80.0%)	
Female	6 (30.0%)	4 (40.0%)	2 (20.0%)	
Age (years old)	61.0 (11.9)	65.3 (7.3)	56.3 (14.0)	0.08
Duration of diabetes	10.6 (11.5)	12.0 (13.5)	9.2 (9.6)	0.60
Height (cm)	159.6 (8.3)	159.1 (10.0)	160.1 (6.7)	0.78
Weight (kg)	65.6 (14.2)	61.1 (11.5)	70.1 (15.8)	0.16
BMI (kg/m2)	25.6 (4.2)	24.2 (3.1)	27.1 (4.8)	0.11
Grip strength (kg)				
Right (kg)	30.2 (9.8)	28.1 (9.6)	32.3 (10.1)	0.37
Left (kg)	29.8 (9.7)	27.6 (8.0)	31.9 (11.6)	0.36
sBP (mmHg)	127 (19)	128 (21)	125 (16)	0.72
dBP (mmHg)	73 (12)	72 (14)	73 (11)	0.75
Chemistry				
HbA1c (%)	9.2 (1.8)	9.3 (2.2)	9.0 (1.4)	0.69
Fasting blood glucose (mg/dL)	148 (39)	152 (36)	144 (45)	0.69
fasting IRI	9.7 (5.5)	10.2 (5.7)	9.2 (5.6)	0.72
fasting CPR (ng/dL)	2.15 (1.28)	2.55 (1.63)	1.76 (0.66)	0.18
Urinary CPR	52.4 (35.0)	55.2 (38.3)	49.6 (33.1)	0.73
UACR (mg/g·Cre)	38.1 (39.7)	42.5 (50.7)	33.7 (26.6)	0.64
Total cholesterol (mg/dL)	159 (33)	157 (20)	161 (42)	0.78
LDL cholesterol (mg/dL)	92 (30)	89 (17)	95 (40)	0.69
HDL cholesterol (mg/dL)	40(12)	39 (16)	41 (8)	0.70
Triglyceride (mg/dL)	137 (77)	145 (60)	128 (93)	0.64
BUN (mg/dL)	17 (6)	18 (8)	15 (4)	0.33
Cre (mg/dL)	0.75 (0.16)	0.72 (0.19)	0.78 (0.13)	0.39
eGFR (mL/min)	78.9 (18.1)	79.9 (21.9)	77.9 (14.6)	0.81
AST (U/L)	30 (23)	35 (30)	25 (13)	0.32
ALT (U/L)	36 (34)	42 (41)	31 (25)	0.48
CVR-R (%)	2.42 (1.09)	1.96 (0.99)	2.89 (1.03)	0.05
R-CAVI	8.3 (1.6)	8.9 (1.3)	7.7 (1.8)	0.08
L-CABI	7.92 (2.15)	8.75 (1.15)	7.10 (2.63)	0.09
R-ABI	1.10 (0.14)	1.12 (0.07)	1.09 (0.19)	0.70
L-ABI	1.10 (0.15)	1.12 (0.11)	1.08 (0.19)	0.62
Diabetec treatment				
OHAs	9 (45%)	5 (25%)	4 (20%)	
GLP-1 RA and OHAs	8 (40%)	3 (15%)	5 (25%)	
Insulin monotherapy	1 (5%)	1 (5%)	0	
Insulin and GLP-1 RA	1 (5%)	0	1 (5%)	
Insulin and GLP-1 RA	1 (5%)	1 (5%)	0	
and OHAs				
study foods				
energy (kcal/day)	target body weight [kg] × energy coefficient [kcal/kg]			
carbohydrates (%energy)	50–60%			
proteins (%energy)	approximately 20%			
fats (%energy)	remaining			
				*n* (%); Mean (SD)

**Table 2:** Results of patient satisfaction questionnaires in each diabetic diet group.

		*n*	Control	D-Allulose	*p*-Value
quantity		107			0.6
	many		10 (19%)	12 (23%)	
	just right		38 (70%)	38 (72%)	
	Not enough		6 (11%)	3 (5.7%)	
seasoning		101			0.322
	Very tasty		4 (8.0%)	3 (5.9%)	
	Delicious		22 (44%)	27 (53%)	
	Neither		21 (42%)	14 (27%)	
	Not tasty		3 (6.0%)	7 (14%)	
coloring		106			0.906
	Very good		4 (7.5%)	4 (7.5%)	
	Good		15 (28%)	18 (34%)	
	usually		33 (62%)	31 (58%)	
	bad		1 (1.9%)	0 (0%)	
smell		107			1
	Good		16 (30%)	15 (28%)	
	not mind about		37 (69%)	38 (72%)	
	stinky		1 (1.9%)	0 (0%)	
Fisher’s exact test					

## Data Availability

The data supporting the findings of this study are available from the corresponding author, K.M., upon reasonable request.
